# Using Machine-Learning Algorithms for Eutrophication Modeling: Case Study of Mar Menor Lagoon (Spain)

**DOI:** 10.3390/ijerph17041189

**Published:** 2020-02-13

**Authors:** Patricia Jimeno-Sáez, Javier Senent-Aparicio, José M. Cecilia, Julio Pérez-Sánchez

**Affiliations:** 1 Department of Civil Engineering, Universidad Católica San Antonio de Murcia, Campus de los Jerónimos s/n, 30107 Guadalupe, Murcia, Spain; 2 Department of Computer Engineering, Universitat Politècnica de València, Camí de Vera, s/n, 46022 Valencia, Spain

**Keywords:** multilayer neural network (MLNN), support vector regression (SVR), water quality, eutrophication, chlorophyll-a, Mar Menor coastal lagoon

## Abstract

The Mar Menor is a hypersaline coastal lagoon with high environmental value and a characteristic example of a highly anthropized hydro-ecosystem located in the southeast of Spain. An unprecedented eutrophication crisis in 2016 and 2019 with abrupt changes in the quality of its waters caused a great social alarm. Understanding and modeling the level of a eutrophication indicator, such as chlorophyll-a (Chl-a), benefits the management of this complex system. In this study, we investigate the potential machine learning (ML) methods to predict the level of Chl-a. Particularly, Multilayer Neural Networks (MLNNs) and Support Vector Regressions (SVRs) are evaluated using as a target dataset information of up to nine different water quality parameters. The most relevant input combinations were extracted using wrapper feature selection methods which simplified the structure of the model, resulting in a more accurate and efficient procedure. Although the performance in the validation phase showed that SVR models obtained better results than MLNNs, experimental results indicated that both ML algorithms provide satisfactory results in the prediction of Chl-a concentration, reaching up to 0.7 R^2^_CV_ (cross-validated coefficient of determination) for the best-fit models.

## 1. Introduction

Coastal lagoons are natural systems with significant environmental and socioeconomic value which occupy approximately 13% of the world’s coastline, representing 5.3% of Europe [1]. They are shallow coastal water bodies isolated from the sea by a barrier, land spit or other similar land feature, but connected to the sea by one or more inlets, through which there is a more or less restricted exchange of water and organisms with the open sea [2,3]. They often exhibit high rates of primary production stimulated by the considerable amounts of nutrients received from surrounding basins [4]. Coastal lagoons are among the most productive ecosystems on the planet, being valuable ecosystems for fishing and aquaculture [5]. In addition, they are generally interesting environments for the development of other human activities, with ideal conditions for nautical sports and swimming, health and entertainment activities, saltworks and the retention and purification of pollutants, among others. However, these natural systems are especially vulnerable to human impacts and the entry of runoff materials [6]. Due to the great variety of transformations and anthropogenic pressures that alter the balance of the coastal lagoon ecosystem, the management of these territories is complex and can result in environmental catastrophes [7]. Among other threats, eutrophication problems associated to human activities have been identified as one of the main causes of water quality impairment of inland and marine waters [8], and is a serious problem worldwide [9]. Eutrophication is a process derived from an increase in the ratio of organic matter supply to an ecosystem, where nutrient enrichment is the most common factor increasing this supply in coastal systems [10]. This nutrient load contributes to an accelerated algal bloom and higher forms of plant life that produce an undesirable disturbance of the equilibrium of the organisms present in the water. The biomass of phytoplankton, represented by chlorophyll-a (Chl-a), is an important indicator to evaluate the state of eutrophication of water bodies [11], and has been studied for decades [12,13]. 

In this context, this study focuses on the Mar Menor lagoon located in the south-eastern Spain as a representative case, due to the serious environmental problems it has suffered in recent decades [14]. Indeed, it is one of the most representative examples of environmental resilience, and has one of the most varied catalogs of anthropic effects on a coastal lagoon in the Mediterranean [15]. In 2016, an unprecedented eutrophication crisis led to serious social and economic problems for this region [15,16,17]. Subsequently, after the “Santa Maria” flood in September 2019, attributed to the meteorological phenomenon known as “gota fría” (cold drop), the ecological degradation of the Mar Menor was aggravated by the massive input of nitrogen, phosphorus and organic matter [18]. As a result of these incidents, the local government approved by decree-law some urgent measures to ensure the environmental sustainability of the Mar Menor area [19,20], and the decree-law on the integral protection of the Mar Menor [21]. These measures include controlling the application of fertilizers and implementing best management practices in the surrounding areas of the coastal lagoon to mitigate water quality problems.

The automatic water quality monitoring is a useful tool to control water quality, especially in critical areas where (1) potential episodes of pollution are expected and/or (2) relevant socioeconomic activities, which require preventive actions are performed. However, to the best of our knowledge, there is no automatic device that accurately measures Chl-a in real time, so Chl-a measurements have to be done in laboratories, which means high latency and high cost. Therefore, it is important to minimize the number of parameters to be measured [22], and it would be very interesting to estimate the water quality parameters values with sufficient precision from the other measured parameters, and to implement and adopt these water quality prediction models that can provide a powerful tool to improve the management of the coastal lagoons. The prediction of surface water quality is a basic task in studies of water resource management, to establish the reasons for the deterioration in water quality and to keep pollution within permissible limits [23,24]. The objective of this study is to obtain a predictive model to calculate the concentration of chlorophyll-a (Chl-a) values from other measured parameters in the Mar Menor Lagoon.

The variables involved in estimating Chl-a in water bodies are complex. In the existing literature, different statistical approaches have been used to determining the Chl-a based on regression analyses. However, these traditional data processing methods generally apply a linear relationship to simplify complex problems, leading to unsatisfactory results because they are not efficient enough to cope with the complicated non-linear relationships between the variables involved [25]. Machine learning (ML) algorithms have demonstrated to be more effective than traditional approaches in determining the water quality [26] as they are very well-suited for predicting nonlinear and complex functions. Previous studies have confirmed the superiority or comparability of ML over traditional approaches in modelling water quality parameters [27,28,29]. ML provides the advantage of performing regressions without the need for a greater knowledge of the water body or the water quality parameters investigated [30]. In particular, Li et al. [31] and Yi et al. [32] applied different types of artificial neural networks (ANNs) to estimate the concentration of Chl-a in 27 lakes in China and in one Korean river, respectively. Another example was presented by Su et al. [25] which developed a structurally simplified hybrid model of the genetic algorithm (GA) and the support vector machine (SVM) for the prediction of monthly concentration of Chl-a in a reservoir of northern China. Nazeer et al. [33] suggested using ML methods, such as ANNs, for more accurate and efficient routine monitoring of coastal water quality parameters, particularly Chl-a, in a coastal area of Hong Kong. Keller et al. [30] concluded that regression models, such as ANN and SVM were very valuable in estimating five water quality parameters, including Chl-a on the river Elbe in Germany. Considering that the SVM and ANN achieved the best result for different water quality parameters in several studies [26,28,29,30], it can be expected that these models will obtain satisfactory Chl-a estimation results in this study.

According to superiority of the ML algorithms, this study has been designed to fulfill the following objectives: (1) To select the specific variables that most related to the Chl-a production using wrapper feature selection algorithms in the Mar Menor lagoon; (2) to develop a predictive model to estimate the Chl-a concentration based on multilayer neural network (MLNN) and support vector regression (SVR) models; (3) to validate the performance of predictive models using different evaluation metrics and identify the best method in estimation of the Chl-a concentration for the Mar Menor lagoon. Several studies have been carried out on the eutrophication process and the water quality parameters of the Mar Menor lagoon [17,34,35,36]. However, to the best of our knowledge, there is no previous research using machine learning models to predict water quality parameters in this lagoon, specifically Chl-a concentration. The rest of the article is organized as follows. Section 2 describes the study area, data collection and the methodology of the study. Section 3 presents the analysis of the data and the results. The discussion of the results is presented in Section 4. Finally, Section 5 summarizes the conclusions.

## 2. Materials and Methods 

### 2.1. Study Area and Data Collection

**Figure 1:**
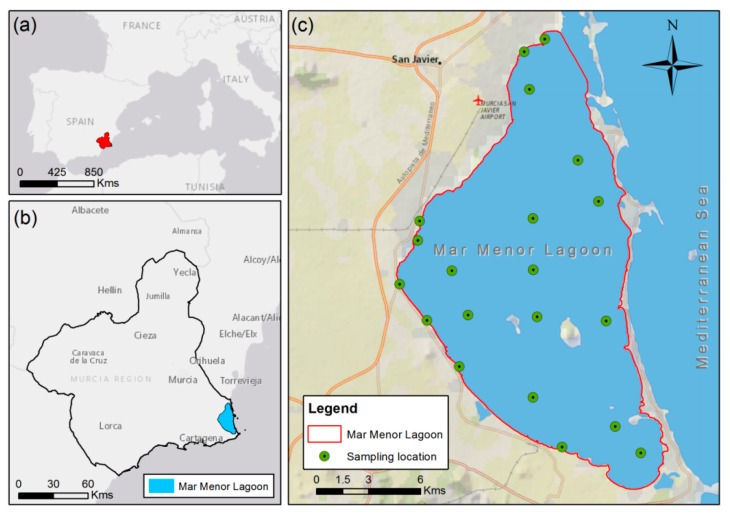
Study site location: (**a**) Location of the Region of Murcia in Spain; (**b**) Location of the Mar Menor lagoon in the Region of Murcia; (**c**) Mar Menor Lagoon and the network of sampling points.

The Mar Menor is the largest hypersaline coastal lagoon in Europe located in the Region of Murcia, a semi-arid area of southeastern Spain (Figure 1). It has an area of 135 km^2^ with 73 km of coastline and houses five islands of volcanic origin in its interior that increase the environmental and landscape value of the area. This lagoon is relatively shallow, with a mean depth of 3.6 m and a maximum depth of 7 m, and is isolated from the sea by a 22 km sand coastal barrier (called La Manga) that is crossed by five channels, causing exchanging its waters with the Mediterranean Sea. 

Tourism and agriculture along the shoreline of the Mar Menor lagoon are a very important activity for the local economy. The drainage area of the lagoon, known as Campo de Cartagena, is a long plain of more than 1600 km^2^ with non-permanent, but abundant surface watercourses that collect the sparse, but intense rainfall [37]. Campo de Cartagena is characterized by intensive agriculture, and its southern zone was a very active mining region for hundreds of years, although this area is currently abandoned [38]. 

Traditionally, the Mar Menor has been characterized by oligotrophic waters and by its great resistance and resilience to the eutrophication process. Its main distinctive feature has been the transparency of its waters, but in the last decade, the lagoon has developed eutrophic characteristics [39]. Changes in agricultural practices in the drainage basin, with the introduction of intensively irrigated crops, have increased inputs with high amounts of nutrient to lagoon during the last decades. The results of several studies [40] delineated the flow transfer from the Campo de Cartagena aquifer to the Mar Menor lagoon with the adverse effect of entry of nitrates and other agrochemical elements from fertilization. These inputs caused a quick increase in the pollution in the lagoon [41], and induced a eutrophication process, leading to a loss of water quality [42,43]. An unprecedented eutrophication crisis in 2016, caused by an abrupt increase in the average concentration of nutrients and chlorophyll, generated an evident change in the quality of the waters, with important increase in the turbidity, change of colour of its waters and loss of transparency with a decrease in the depth of visibility of the Secchi disc to less than 1 m. These caused a great alarm both in environmental circles and in the tourism sector with important socioeconomic consequences [15,16,17]. After considerably reducing the supply of nutrients from agricultural sources, the system began a rapid recovery, which was evident in the spring and summer of 2018 [17]. In September 2019, surface runoff water from the drainage basin that flowed into the Mar Menor, due to a meteorological phenomenon of the cold drop, caused a strong increase in chlorophyll levels, even above the maximum reached in 2016. The huge amounts of nitrogen, phosphorus, organic matter and sediments washed away by runoff were the main factors for the primary productivity [18]. 

In this context, urgent action is needed to reduce the entry of nutrients and other pollutants into the lagoon. This need is further reinforced by the declaration of the Mar Menor basin as a nitrate pollution vulnerable zone (91/676/EEC), the declaration of the lagoon as a sensitive area subject to eutrophication in application of the Urban Wastewater Directive (91/271/ECC) and the application of the Water Framework Directive (2000/60 EC), which obliges to achieve and maintain the Good Status of all bodies of water and requires the monitoring and management of the ecological status of surface waters by all Member States, including coastal and transitional ones. In addition, the Mar Menor and associated wetlands has been protected by a series of regional national and international rules and resolutions [9,42]: (1) The Ramsar List of Wetlands of International Importance, (2) Special Protected Areas of Mediterranean Interest, (3) Specially Protected Area under the EU Wild Birds Directive, and (4) the Nature 2000 Network as a Site of Community Importance. For these reasons, the conservation of the Mar Menor requires integrated and sustainable planning and management of its basin.

In this study, the daily data on water quality in the Mar Menor were obtained from oceanographic campaigns carried out by the local government, Comunidad Autónoma de la Región de Murcia (CARM), and the information is available on the Mar Menor information service website (http://www.canalmarmenor.es/web/canalmarmenor/parametros). The data sets consisted of 10 physical and chemical parameters measured at 20 sampling points throughout the lagoon (Figure 1) from September 2017 to December 2018. Only 126 daily data were available to date. These parameters were Chl-a, water temperature (T), pH, suspended solids (SS), turbidity (TU), Secchi Disk depth (SD), salinity (S), dissolve oxygen (DO), total nitrogen (TN) and total phosphorus (TP). 

### 2.2. Modeling Approaches and Feature Selection (FS)

The different steps followed in this study are as follows: (1) Selection of the input parameters by FS algorithms; (2) building a predictive model (MLNN and SVR) using the selected input scenarios for model learning; and (3) evaluating the predictive models using several metrics to validate the performance.

#### 2.2.1. Multilayer Neural Network (MLNN)

The ANN is a massively parallel-distributed information-processing system that attempts to simulate the functioning of brain neurons using a network of artificial neurons organized into layers [44]. The network receives a stimulus and transforms this input into an output signal through a transfer function. The ANN model is an appropriate technique for modelling because of its capability to assign significance to input parameters and to map the inputs to the outputs when the relationships between the variables of the underlying physical processes are complex or unknown [45]. These neural networks are a non-linear modeling tool that can manage a large number of inputs (independent variables) to determine one or more outputs (dependent variables) [46]. 

There are many types of ANNs for different applications. In a feedforward network, the direction of information flow between nodes or neurons is from the input to the output layer, and each node in a layer is connected to each of the nodes in the next layer, but not to those in the same layer [47]. The nodes are connected to other nodes by links which have an associated weight that represents its connection strength and stores the knowledge of the network [48]. The mathematical operation of a node can be summarized according to the following Equation (1):

(1) $$y_{j}\;=\;f\;\left(\overset{n}{\underset{i=1}{\sum }}x_{i}\cdot w_{i}\;-\;b_{j}\right)$$

where y is the output of a neuron *j*, *f* is an activation function, x*_i_* is an input of the vector of inputs (*I =* 1, 2, …, *n*), w*_i_* is the weight associated with the connection link through which the input x*_i_* arrives to current neuron *j* from a neuron in the preceding layer and b*_j_* is a bias associated with neuron *j*. Therefore, the connection weights, biases, and transfer functions parameterize the mathematical relationship between inputs and outputs of the network [49]. These weights and biases need to be adjusted in the training process of the networks to minimize the model error. 

To estimate the Chl-a level in the Mar Menor, MLNNs were developed based on the feedforward back-propagation method. These networks consist of a number of nodes organized in an input layer, one or more hidden layers and an output layer. In the hidden layer, which is the most important part of the ANN, the nodes receive the signals only from neurons in the previous layer and process data. This processed data was fed to the output layer where the output is calculated. 

**Figure 2:**
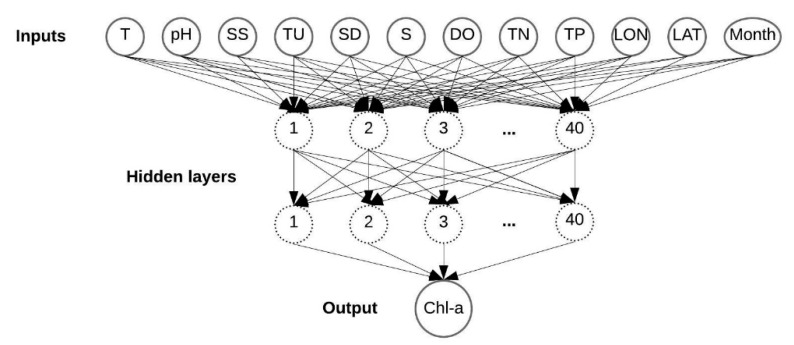
An multilayer neural network (MLNN) structure developed for predicting the concentration of chlorophyll-a (Chl-a) with two hidden layers and 12 variables as inputs (water temperature (T), pH, suspended solids (SS), turbidity (TU), Secchi Disk depth (SD), salinity (S), dissolve oxygen (DO), total nitrogen (TN), total phosphorus (TP), latitude (LAT), longitude (LON) and month).

The script of the MLNN models was implemented in MATLAB® software (version 8.2.0.701 (R2013b), The Mathworks, MA, USA). In this study, one or two hidden layers were considered, with a sigmoid function in the hidden layers and one output layer with a linear function. Specifically, the logistic sigmoid (logsig) and hyperbolic tangent sigmoid (tansig) functions were tested to obtain better results with respect to non-linearity of this process. Figure 2 describes an example of an MLNN used in this study. 

The input layer of the neural networks contained as many nodes as there were input parameters and the output layer contained only one node. Numbers of layers (1 or 2) and a number of nodes (between 5 and 40) in the hidden layers were tested and determined using trial and error. All these adjustable parameters were tested to yield a good performance of the network. The mean squared error (MSE) was used to define the network error and minimized during the training process. Four training algorithms, which are the fastest and most commonly adopted in MLNN training [50,51], were tested: Levenberg-Marquardt (LM) backpropagation, BFGS quasi-Newton backpropagation (BFG), resilient backpropagation (RP) and conjugate gradient backpropagation with Fletcher-Reeves updates (CGF).

#### 2.2.2. Support Vector Regression (SVR)

SVM is a supervised machine learning technique that is executed following the structural risk minimization principle and statistical learning theory. The SVM algorithm transforms the original input space into a higher dimensional feature space to find an optimum hyper plane of separation [52]. The SVM model is commonly used in classification problems, but can be easily adopted in regression problems. In fact, the originally developed method was extended into SVR by introducing a ε–insensitive loss function for application in regression case studies. The theory of SVR development is available in Vapnik et al. [53]. In this study, the SVR models were developed in the R software and a radial basis function implemented in the “caret” package [54,55] was selected as kernel function and used to estimate the concentration of Chl-a. There are two tuning parameters in this model, the scale function (σ) in the radial basis function (see Equation (2)) and the cost value (C) used to control the complexity of the decision boundary. The application of an adaptive cross-validation resampling technique [56] provides a computationally efficient way to identify these parameters for each specific model of SVR.

(2) $$K\left(x,x_{i}\right)=exp\left(-\sigma \|x-x_{i}\|{}^{2}\right)$$

where $x_{i}$ is the input vector with $x\in R^{n}$. 

#### 2.2.3. Assessing Model Performance

In an effort to check any overfitting, the 5-fold cross-validation was performed. The data set was divided into five subsets—four subsets were used for training, and the remaining one for validation. The holdout method was repeated five times, and the regression results of 5-fold cross-validation were averaged and presented as overall testing results of the models. The performance of the models was evaluated using three indicators calculated from predicted and measured data. Overall performance was analyzed with cross-validated coefficient of determination (R^2^_CV_), the proportion of systematic error in the overall with cross-validated root mean squared error (RMSE_CV_) and overall errors with cross-validated mean absolute error (MAE_CV_). These statistics are defined in Table 1.

#### 2.2.4. Feature Selection (FS)

Machine learning algorithms (MLAs) are generally applied from a set of training instances in which each instance is described by a feature vector (input parameters), and target feature (output parameters) expressed as a continuous value in regression problems [57], where the main objective of predictive modeling is to maximize accuracy [58]. To estimate a parameter of water quality can use all available features, or select a smaller number of them. This can result in the inclusion of too few or too many inputs to the model, both of which are undesirable [59]. To address this issue, an FS stage had been considered in this study to eliminate redundant data. FS is a process that selects a subset of features from the original set, so that the feature space is optimally reduced according to a certain criterion. The goal of reducing the dimensionality of the feature space in ML is to speed-up the operation of the learning algorithm to improve predictive accuracy and enhance the comprehensibility of the learning results [58]. There are many parameters that influence the concentration of Chl-a. This study performed an FS to determine the appropriate input vectors of a prediction model with three hybrid methods: Wrapper algorithms (recursive feature elimination (RFE), GA and simulated annealing (SA)) combined with random forest (RF) and SVR. The wrapper algorithms evaluate multiple models using procedures that add and/or remove predictors to find the optimal combination that maximizes model performance. In essence, wrapper methods are search algorithms that treat predictors as inputs and use model performance as output to optimize. These procedures, as implemented in the “caret” package [55] of the R software. These hybrid methods contained two steps. In the first, RFE, GA or SA is used to select a subset of features from all features. Moreover, in the second step, RF or SVR is used to assign a weight of importance to each feature included in the selected subset. 

RFE uses a backwards elimination approach, starting with all features and eliminating one at a time. At each step, the feature that is considered least useful for prediction is removed, and the overall performance of the predictor is reevaluated through cross-validation [60]. GA is based on Darwin’s “survival of the fittest” and was applied to search for the better subset of features. The subset of features was selected based on the current population (i.e., subset of features) through crossover, mutation, and selection according to the fitness function. SA algorithm is a global search method that makes small random changes to an initial candidate solution [55].

## 3. Results 

### 3.1. Inputs Selection 

The basic descriptive statistics of the input variables used in this study are presented in Table 2. The X_max_, X_min_, X_mean_, St. Dev., C.V. and correlation coefficient (CC) denote the maximum, minimum, mean, standard deviation, coefficient of variation of the data and correlation coefficient with Chl-a, respectively.

The CC is used to explore the dependences between the variables. Each CC measures the degree of the linear relationship of the variable Chl-a with the other parameters. The CCs marked as bold were significant at *p*-level < 0.05. 

In this study, eight scenarios with different input combinations of the variables are tested for estimating of Chl-a concentration values in the Mar Menor lagoon. As water quality can change spatially and seasonally the variables month, latitude (LAT) and longitude (LON) are added to the set of variables. The first input scenario (M1) considered all parameters as inputs without feature selection. The second scenario (M2) included only the most highly correlated parameters. The other input scenarios (M3–M8) are extracted through the wrappers algorithms. Summarized results of feature selection ranked by importance using wrappers are shown in Table 3. 

### 3.2. Model Comparison and Prediction Accuracy

The MLNN and SVR models with eight input scenarios described in Table 3 were developed to simulate the Chl-a concentration. The eight versions of each model represent eight substantially different chlorophyll models, due to the different combinations of variables used as predictors. Different ML modes are compared based on the three statistical indices obtained in 5-fold cross validation: R^2^_CV_, RMSE_CV_ and MAE_CV_**.** These performance measurements in the training and testing phase are summarized in Table 4 and Table 5 for MLNNs and SVRs, respectively. Different parameters are tried for each MLNN and SVR model and the best ones; i.e., with the minimum RMSE_CV_ in the testing phase, are selected for each input scenario.

The best results are obtained with a network structure of two hidden layers with logsig transfer functions for the eight versions of the MLNN model. The BFG algorithm is the most efficient in all versions except for the MLNN-M5, MLNN-M7 and MLNN-M8 where the RP algorithm defeats them by a slight margin. The comparative results between the eight versions of the MLNN model reveal that the MLNN-M5 with nine inputs selected by GA-RF wrapper algorithm yielded the best accuracy among all the developed MLNN models in term of higher R_CV_ and lower RMSE_CV_ and MAE_CV_ values for the training (R_CV_ = 0.74, RMSE_CV_ = 0.73 mg/m^3^ and MAE_CV_ = 0.49 mg/m^3^) and testing phase (R_CV_ = 0.62, RMSE_CV_ = 0.89 mg/m^3^ and MAE_CV_ = 0.66 mg/m^3^). MLNN-M5 is only outperformed by the MLNN-M4 model in terms of lower MAE_CV_ for the testing phase. With also nine inputs, the MLNN-M4 model is the second most accurate model with a performance close to the best. 

Regarding the SVR models, the results of parameter optimization of SVR models indicated that the optimal C values are between 0.58 and 4.20, and the optimal σ values in radial basis kernel function are between 0.07 and 0.30. The SVR-M5 model obtained the best results in the training phase (R_CV_ = 0.58, RMSE_CV_ = 0.92 mg/m^3^ and MAE_CV_ = 0.64 mg/m^3^) and the SVR-M4 model in the test phase (R_CV_ = 0.68, RMSE_CV_ = 0.81 mg/m^3^ and MAE_CV_ = 0.56 mg/m^3^). 

Models that used M4 scenario with nine input variables (SD, T, SS, S, TP, pH, TN, LON and TU) and models that used M5 input scenario with also nine inputs (LON, T, pH, SS, TU, SD, S, DO and TP), obtain an accuracy very similar for both models (MLNN and SVR). The difference between these models is that M5 uses the DO variable instead of the TN variable.

## 4. Discussion

The Chl-a concentrations had a significant correlation with water quality variables as SS, SD, S and TP in the study area, but the weakness of these correlations indicates that the use of traditional regression methods in modeling such a complex process is irrelevant, so there is a great need to use more powerful techniques [61].

The results of the feature selection (see Table 3) are similar to those obtained in other studies. Although the influence variables of Chl-a were different in several research works, the TP and TN concentration generally were among the main variables. For example, Palani et al. [62] applied the ANN model with location variables (LON and LAT), PO_4_, DO and T as the explanatory variables to predict Chl-a concentration. Li et al. [31] selected the concentration of PT and TN, T, SD, and DO among the most influential input variables for Chl-a using a genetic algorithm optimized back-propagation neural network. Furthermore, Kuo et al. [63] defined the Chl-a model by the input of month, T, pH, SD, SS, PO_4_ and NO_3_. Moreover, GA_RF was the algorithm that selected more features with ten of the twelve features selected. In contrast, the algorithm that selected the least characteristics is SA_SVM by selecting only seven. LON is selected by all the algorithms, being the only variable common to all the scenarios. The month factor which shows that the Chl-a changes seasonally is only selected as input in one case.

According to the average performance of the models (Table 4 and Table 5), MLNN and SVR models performed reasonably. Considering that the length of the data and its quality have a significant influence on the modelling process [64], the results can be considered acceptable and promising, considering that only 126 daily data are available. 

To conclude, the SVR models perform better than the MLNN models for all input scenarios in the testing phase except for the M2 scenario, and the best-fit model is the SVR-M4. In addition, MLNNs have the disadvantage of having to be trained for each problem to obtain the optimal architecture, and this requires great computational resources, greater than for SVR models [65]. The worst results are obtained by the models with the M2, M6, M7 and M8 scenarios. M6, M7 and M8 scenarios did not include as input the variables SS, S, SD or TP that are significantly correlated with Chl-a or other variables, such as T or pH. However, when only the most correlated variables are included (M2 scenario), the models also perform poorly. This fact could support the conclusion drawn by Maier et al. [59] that using a linear approach to identify which of the potential input variables have a significant relationship with the model output is not appropriate for the development of ANN models. Consequently, there is a need to adopt other input selection approaches as FS using algorithms. On the other hand, the FS managed to improve the results with respect to scenario M1 (without the selection of feature) in three scenarios (M3, M4 and M5). The wrapper algorithm that provides the lowest results was the SA. However, FS contribute to reducing the modeling time by reducing the number of input parameters in all cases.

## 5. Conclusions

The current significant ecological deterioration of the Mar Menor lagoon makes it necessary to improve the understanding of the interaction between the water quality parameters of the coastal lagoon to adopt integrated and sustainable management strategies. This coastal lagoon has recently suffered a eutrophication crisis. Chl-a concentration is one of the most important indicators of the existence and degree of eutrophication in water bodies.

The results confirmed the importance and usefulness of intelligent modeling as a rapid, easy to operate and not expensive tool. The MLNN and SVR models have great potential in modelling complex and heterogeneous systems, such as coastal lagoons. The capabilities of these models in estimation of the concentration of Chl-a have been investigated using water quality inputs, such as T, pH, SS, SD, S, DO, TU, TN and TP monitored at different stations in the Mar Menor lagoon. Both algorithms with eight input combinations and the cross validation method are employed in this study. Comparison between simulated and observed data showed the effectiveness of the models, where most of the values of RMSE_CV_ and MAE_CV_ are small, and most of R^2^_CV_ were close to 0.7 for the best models. Finally, the better model is selected based on the performance measurements. Average performances of the models indicated that the SVR with nine input variables (SD, T, SS, S, TP, pH, TN, LON and TU) is more capable than the alternative models in estimating the values of Chl-a concentration. 

The main finding in this study revealed that the MLNN and SVR models could be used as a successful tool to estimate Chl-a concentration based on other measured parameters and these estimation results are useful for the coastal lagoon quality management. This can be especially useful in estimating other parameters, which are difficult to measure, as an alternative way for monitoring and assessment water quality in real time based on other water quality monitoring data from the studied area. Furthermore, MLNN and SVR models can also be used to simulate the future state of the study area using several scenarios for water quality parameters. The limitations of this study include its limited data set. The results obtained in this work could be improved in future work if more on-site measured data were available. Therefore, it is strongly recommended that the sectors related to water quality in the Mar Menor lagoon focus on improving monitorization of environmental variables and sharing the available data. Additionally, other algorithms should be tested in future work.

## Figures and Tables

**Table 1:** Performance metrics.

Performance Metric	Equation	Range
Cross-validated coefficient of determination (R^2^_CV_)	$\frac{{\left[\sum_{i=1}^{n}\left(O_{i}-\bar{O}\right)\cdot (E_{i}-\bar{E})\right]}^{2}}{{\left[{\left[\sum_{i=1}^{n}{\left(O_{i}-\bar{O}\right)}^{2}\right]}^{0.5}\cdot {\left[\sum_{i=1}^{n}{\left(E_{i}-\bar{E}\right)}^{2}\right]}^{0.5}\right]}^{2}}\;$	[0, 1]
Cross-validated root mean squared error (RMSE_CV_)	$\sqrt{\frac{\sum_{i=1}^{n}{\left(O_{i}-E_{i}\right)}^{2}}{n}}$	[0, ∞]
Cross-validated mean absolute error (MAE_CV_)	$\frac{\sum_{i=1}^{n}\left|O_{i}-E_{i}\right|}{n}$	[0, ∞]

*O_i_* is the *ith* observed data, $\bar{O}$ is the mean of the observed data, *E_i_* is the *ith* estimated data, $\bar{E}\;$ is the mean of the estimated data and *n* is the total number of observations.

**Table 2:** Daily statistics of the water quality parameters between September 2017 and December 2018 in the Mar Menor Lagoon.

Parameters (Units) ^1^	X_max_	X_min_	X_mean_	St. Dev. ^2^	C.V. ^3^	CC ^4^
Chl-a (mg/m^3^)	7.50	0.13	2.02	1.43	0.71	**1.00**
T (°C)	27.70	11.00	20.09	6.41	0.32	–0.08
pH	8.46	7.83	8.17	0.12	0.01	–0.02
SS (mg/l)	35.35	5.00	8.64	4.94	0.57	**0.22**
TU(NTU)	24.00	0.50	2.82	3.37	1.19	0.002
SD (m)	6.50	0.30	2.39	1.63	0.68	**–0.55**
S (PSU)	46.38	41.86	44.21	0.95	0.02	**–0.35**
DO (mg/l)	8.12	4.25	6.55	0.80	0.12	–0.17
TN (mg N/l)	8.84	0.16	0.59	0.80	1.35	0.09
TP (mg P/l)	0.07	0.01	0.01	0.01	0.70	**0.25**

^1^ T is water temperature, SS is suspended solids, TU is turbidity, SD is Secchi Disk depth, S is salinity, DO is dissolve oxygen, TN is total nitrogen and TP is total phosphorus; ^2^ St. Dev.: Standard deviation; ^3^ C.V.: Coefficient of variation (St. Dev./Xmean); ^4^ CC: Correlation coefficient with Chl-a.

**Table 3:** Summarized results of feature selection using wrapper algorithms.

Algorithm ^1^	N. of Features Selected	Features Selected ^2^	Input Scenario
Without feature selection	12	T, pH, SS, SD, S, DO, TU, TN, TP, LON, LAT, month	M1
The most highly correlated features	4	SD,S,TP,SS	M2
RFE_RF	9	SD, S, SS,TN, T, pH, DO, TP, LON	M3
RFE_SVM	9	SD, T, SS, S, TP, pH, TN, LON, TU	M4
GA_RF	9	LON, T, pH, SS, TU, SD, S, DO, TP	M5
GA_SVM	10	month, LAT, LON, T, pH, TU, SD, DO, TN, TP	M6
SA_RF	8	LAT, LON, pH, TU, SD, S, DO, TN	M7
SA_SVM	7	LON, T, SS, TU, S, TN, TP	M8

^1^ Wrapper algorithms: recursive feature elimination (RFE), genetic algorithm (GA) and simulated annealing (SA) combined with random forest (RF) or support vector regression (SVR). ^2^ LAT is latitude and LON is longitude.

**Table 4:** Performance of Chl-a estimation from MLNN models based on eight different input scenarios obtained in 5-fold cross validation.

Model-Input Scenario	Architecture [I–H1–H2–O] ^1^	Training Phase			Testing Phase		
		R^2^_CV_	RMSE_CV_ (mg/m^3^)	MAE_CV_ (mg/m^3^)	R^2^_CV_	RMSE_CV_ (mg/m^3^)	MAE_CV_ (mg/m^3^)
MLNN-M1	[12–16–27–1]	0.63 ± 0.14	0.85 ± 0.21	0.59 ± 0.21	0.53 ± 0.16	0.98 ± 0.31	0.70 ± 0.22
MLNN-M2	[4–12–17–1]	0.62 ± 0.11	0.88 ± 0.16	0.61 ± 0.09	0.52 ± 0.17	0.95 ± 0.32	0.71 ± 0.17
MLNN-M3	[9–31–23–1]	0.67 ± 0.17	0.80 ± 0.25	0.54 ± 0.15	0.60 ± 0.23	0.89 ± 0.41	0.66 ± 0.24
MLNN-M4	[9–32–39–1]	0.72 ± 0.07	0.76 ± 0.14	0.50 ± 0.06	0.61 ± 0.16	0.89 ± 0.35	0.63± 0.18
MLNN-M5	[9–40–39–1]	0.74 ± 0.09	0.73 ± 0.17	0.49 ± 0.13	0.62 ± 0.13	0.89 ± 0.26	0.66 ± 0.14
MLNN-M6	[9–40–33–1]	0.72 ± 0.13	0.74 ± 0.20	0.51 ± 0.20	0.55 ± 0.21	0.99 ± 0.34	0.76 ± 0.19
MLNN-M7	[8–39–16–1]	0.72 ± 0.08	0.78 ± 0.15	0.57 ± 0.13	0.54 ± 0.16	0.96 ± 0.32	0.71 ± 0.18
MLNN-M8	[7–23–26–1]	0.72 ± 0.08	0.74 ± 0.14	0.47 ± 0.10	0.53 ± 0.11	0.98 ± 0.24	0.68 ± 0.13

^1^ I is the number of neurons in input layer; H1 and H2 is the number of neurons in hidden layer 1 and hidden layer 2; O is the number of neurons in output layer.

**Table 5:** Performance of Chl-a estimation from SVR models based on eight different input scenarios obtained in 5–fold cross validation.

Model-Input Scenario	Model Parameters	Training Phase			Testing Phase		
		R^2^_CV_	RMSE_CV_ (mg/m^3^)	MAE_CV_ (mg/m^3^)	R^2^_CV_	RMSE_CV_ (mg/m^3^)	MAE_CV_ (mg/m^3^)
SVR-M1	σ = 0.07 C = 3.31	0.53 ± 0.05	0.99 ± 0.07	0.70 ± 0.03	0.56 ± 0.09	0.85 ± 0.26	0.62 ± 0.14
SVR-M2	σ = 0.24 C = 0.58	0.45 ± 0.06	1.12 ± 0.08	0.80 ± 0.03	0.49 ± 0.19	0.98 ± 0.34	0.71 ± 0.16
SVR-M3	σ = 0.13 C = 2.54	0.56 ± 0.05	0.96 ± 0.12	0.66 ± 0.05	0.65 ± 0.11	0.82 ± 0.30	0.58 ± 0.16
SVR-M4	σ = 0.10 C = 2.59	0.58 ± 0.06	0.94 ± 0.10	0.66 ± 0.04	0.68 ± 0.10	0.81 ± 0.32	0.56 ± 0.17
SVR-M5	σ = 0.10 C = 3.01	0.58 ± 0.05	0.92 ± 0.09	0.64 ± 0.04	0.67 ± 0.09	0.82 ± 0.30	0.57 ± 0.18
SVR-M6	σ = 0.10 C = 4.20	0.52 ± 0.09	0.98 ± 0.11	0.69 ± 0.06	0.61 ± 0.16	0.86 ± 0.31	0.61 ± 0.15
SVR-M7	σ = 0.13 C = 3.38	0.51 ± 0.10	1.03 ± 0.15	0.72 ± 0.08	0.57 ± 0.20	0.90 ± 0.37	0.64 ± 0.21
SVR-M8	σ = 0.30 C = 2.63	0.47 ± 0.05	1.06 ± 0.07	0.74 ± 0.04	0.54 ± 0.13	0.94 ± 0.21	0.66 ± 0.13
